# How Cultural Involvement in Different Brand Types Influences Willingness to Pay Premium: The Mediating Role of Brand Happiness

**DOI:** 10.3390/bs14100908

**Published:** 2024-10-08

**Authors:** Zhimin Zhou, Yuan He, Yufeng Xie, Shafaqat Mehmood

**Affiliations:** 1 College of Management, Shenzhen University, Shenzhen 518060, China; mnizzm@szu.edu.cn (Z.Z.); 2150137001@email.szu.edu.cn (Y.H.); 2 China Center for Special Economic Zone Research, Shenzhen University, Shenzhen 518060, China; 3 School of Business, Sun Yat-sen University, Guangzhou 510275, China; xieyf57@mail2.sysu.edu.cn

**Keywords:** brand culture, rand enriching benefits, willingness to pay a premium, brand type, brand happiness

## Abstract

This study examines the impact of cultural involvement on brand enriching benefits and consumers’ willingness to pay a premium (WPP). Additionally, it explores the underlying mechanisms behind this impact. A partial least squares structural equation model was used to analyze the data collected from a pretest (N = 70) and an online survey (N = 1053) conducted in China. The moderating effect of brand type was examined using a multi-group analysis. The results indicate that cultural involvement has a significant and positive impact on brand enriching benefits, consequently enhancing consumers’ WPP. Additionally, brand happiness mediates the relationship between brand enriching benefits and WPP. Through a multi-group analysis, the impact of brand culture on brand equity is found to be more pronounced in functional brands compared with symbolic brands. Additionally, high perceived quality facilitates the transformation of brand enriching benefits into WPP. Managers value the ability of a brand culture to create brand equity and implement differentiation strategies based on different brand types. It is critical for managers to use a culture and its rich benefits wisely and to consider consumer happiness in the brand experience. Although brand culture and its impact on brands are crucial, previous studies have only focused on qualitative analyses, while empirical research on the direct link between brand culture and equity has been lacking. This study empirically tests a conceptual model that elucidates the relationship between brand cultural involvement, brand enriching benefits, brand happiness, WPP, brand type, and perceived quality. It extends previous research and offers suggestions for academics and brand managers interested in effectively leveraging brand culture for brand building.

## 1. Introduction

Whether a time-honored or new consumer brand is being built, marketing practitioners try to integrate cultural features into the brand [1]. Cultural features add a wide range of subtle aspects that help build upon a culture’s identity, such as customs, language, festivals, food and cuisine, art, and architecture. The continued leadership of brands such as Coca-Cola and Nike comes from their cultural relevance with these brands having become iconic cultural brands [2]. For instance, the Chinese perfume brand ‘To Summer’ uses natural and cultural fragrances evocative of East Asian culture, and it names many of their products after elements of East Asian culture, which are accompanied by a narrative about the product’s naming inspiration. Therefore, understanding the interaction between a brand and culture elements is important for building time-honored or new consumer brands [3].

Culture plays an important role in brand relationship research and consumer behavior research. Many studies have explored the basis for the formation of brand culture, i.e., a certain cultural value or cultural heritage. For example, Holt [4] argued that brand culture comes from the interaction of corporate history, dominant social culture, users, etc. Banerjee [5] constructed a Brand–Culture Fit Model to combine brand and cultural heritage to find commonalities upon which to build a brand’s culture. Other researchers explored the path of brand culture formation. Brand culture is the link between brand communities, and the culture in online brand communities is usually created by community members through a variety of methods [6,7,8]. In the current study, brand culture is defined as the inclusion of cultural elements in a brand. These cultural elements are the epiphenomenal elements of the culture through which people must perceive the meaning of culture. Cultural involvement, an intangible state of motivation and interest toward products with attractive characteristics [9,10], is the extent to which consumers are aware of, interested in, and prefer a certain culture [11]. This study considers the consumer’s interest, preferences, and affection for the brands’ culture to be the cultural involvement of the brand.

What does brand culture bring to consumers and brands? By creating a strong brand culture, companies can foster a sense of connection and belonging among their customers [7], leading to positive marketing responses [12] and positive consumer responses (e.g., brand loyalty and willingness to pay a premium) that drive firm performance [13]. Wiedmann et al. [14] took the cultural value of a brand as an important component in the concept of brand heritage and examined the effects of brand heritage on brand image, brand trust, brand loyalty, consumer satisfaction, and consumers’ willingness to pay a premium. The results showed the cultural meaning of a brand can promote strong self-brand connection and generate brand equity.

From the consumer’s perspective, brand equity, known as “customer-based brand equity” (CBBE), refers to consumers’ ability to differentiate between brands, which is demonstrated in their brand knowledge during marketing communications [15]. Brand equity embodies the values of the brand. Willingness to pay a premium (WPP) is the primary aspect of brand equity [16]. Previous research has shown that WPP is the result of effective brand management [17], as it reflects a brand’s ability to command a higher price than its competitors and is considered important for all types of brands [18]. The concept of willingness to pay a premium can be understood as the highest price level that consumers are willing to pay for goods or services [19]. Brand happiness is also a promising new brand equity and refers to the positive emotions and experiences consumers have associated with a particular brand [20]. Brand happiness includes four constructs: joy (emotions that reflect consumers’ exuberance toward brands), vigor (a high degree of activation and vitality), pride (feelings of self-enhancement in relation to the brand), and serenity (emotional harmony and balance regarding the brand). In addition, brand happiness is the antecedent of consumers’ actual behavior (brand loyalty, WPP, word of mouth, brand evangelism, etc.) [21,22,23].

On the one hand, brand experience can increase consumers’ willingness to pay a premium [24]. Perceived brand interactivity and involvement directly impact social media consumer brand engagement, which is strongly related to the behavioral aspect of brand loyalty [25]. On the other hand, culture can give a brand more in-depth and quick assessments of the market through marketing communications [4], and brand managers connect brands to culture in the brand experience. Nonetheless, not much relevant academic research has been conducted, especially empirical studies on the relationship between brand culture and brand equity. Table 1 summarizes the current research related to brand culture.

As a result, this study aims to answer the following research questions:

**RQ1.**  *How do brands gain cultural meaning and increase consumers’ willingness to pay a premium?*

**RQ2.**  *What are the underlying mechanisms (moderating and mediating effects) of the relationship?*

This paper is structured as follows: Section 2 discusses the theoretical background, theoretically explaining the fundamental areas of study and justifying the effectiveness of the framework of the theory. Section 3 establishes the hypothesis and the interplay among the paper’s concepts. Section 4 describes the method used in this study. Section 5 presents the results of this study. Section 6 summarizes the main findings, theoretical contributions, management implications, limitations, and suggestions for future research.

## 2. Theoretical Background

Brand culture theory, which falls under consumer culture theory (CCT), clarifies the importance of culture for brands. Consumer culture theory centers on the cultural symbolism of consumption [31], and brand culture theory emphasizes the cultural meaning and impact of brands. The meaning of brand culture is not uniform because culture itself can be understood in many ways. The mainstream view is to understand a brand culture from the perspective of its cultural meaning and symbolism.

According to the theory of meaning transfer, managers are able to transfer cultural meanings into brands, and consumers are able to facilitate this process of transferring cultural meanings [32]. Fournier and Alvarez [29] commented on how cultural meanings are integrated into brands and give brands a relational capacity to resonate with people, giving new and different meanings to the brand. Steenkamp [33] explored the possibility of positioning a brand as a symbol of a certain culture. Gürhan-Canli et al. [34] elucidated the impact of culture on consumer–brand interactions. Holt [4] argued that brand culture is a collection of relevant stories told by the company about the brand, the dominant culture, the influencing forces, and the customers. In this study, we highlight consumers’ cultural perceptions of the brand and the resulting value-added capabilities. Accordingly, this study introduces the concepts of brand cultural involvement (BCI) and brand enriching benefit (BEB) to represent this process.

Research has shown that consumers’ attitudes and preferences toward specific cultures within a brand determine their attitudes and emotions toward the brand [35]. In tourism research, increased involvement resulted in perceived higher destination brand equity, and highly engaged cultural tourists are more likely to enjoy the destination [11]. Tourists who are more involved in the destination culture became more satisfied with the experience and developed a stronger brand attachment [36]. The antecedent for brands to strongly associate culture with their brand comes from consumers being aware of and enjoying the culture itself, which is denoted as brand cultural involvement.

When consumers purchase products that are consistent with their personal beliefs or preferences, it can provide enriching benefits [37,38]. Brand enriching benefits encompass the cultural resonance that a brand has with consumers in terms of how much the brand enhances its consumers’ self-perception [39]. On the one hand, the enriching benefits of a brand focuses on the brand itself, which is not only the value that a brand may have but also an identity and self-expression [40]. On the other hand, the enriching benefits of a brand underscores consumer appeal and represents the relations between brand equity and individual consumers [41]. Intrinsic motivation such as self-expression influences customers’ brand engagement, like online communities, leading to actionable behavior such as brand advocacy [42]. Park, MacInnis, and Eisingerich [38] proposed to increase brand enriching benefits to cultivate customer loyalty behavior.

According to Keller and Lehmann [12], the financial outputs of a brand are derived from the consumer’s mind. The presence of brand enriching benefits may make consumers happy [43]. As we mentioned before, brand happiness is the pleasant experience and spiritual satisfaction that consumers perceive through contact with brands. In addition, brand happiness is the antecedent of consumers’ actual behavior (brand loyalty, WPP, word of mouth, brand evangelism, etc.) [21,22,23]. Therefore, this study analyzes the impact of brand culture on brand equity to reveal the impact of brand cultural involvement (BCI) on willingness to pay a premium (WPP) and the mediation effect of brand enriching benefit (BEB) and brand happiness. Aside from that, this study further explores the moderating role of brand type and perceived quality in this nexus.

## 3. Hypothesis Development

### 3.1. Brand Cultural Involvement and Brand Enriching Benefits

Consumers’ awareness of, interest in, and preference for a certain culture is cultural involvement [11], which determines their attitudes and emotions toward the brand [35]. Through strategies such as emphasizing brand heritage and brand history [44], establishing a link between the brand and the culture of origin [14], and marketing communication in conjunction with culture [45], consumers develop a perception that the brand is associated with a specific culture.

According to the theory of meaning transfer, cultural meaning moves first from the environment to consumer goods and then from these goods to the individual [29,32,46]. Brands gain cultural significance from culture, which is a brand culture that resonates with individual consumers [47]. Consumers feel inspired when the brands they buy and consume from have cultural elements they personally love, which can provide enriching benefits [37,38]. With this, the following hypothesis is presented:

**Hypothesis** **(H1).** *BCI positively influences BEB.*

### 3.2. Brand Enriching Benefits and Willingness to Pay Premium

The cultural significance of a brand enriches the consumer’s brand experience [29]. Pleasant experiences are essential in promoting brand equity. Dwivedi, Nayeem, and Murshed [24] argue that brand experience can increase consumers’ willingness to pay a premium. A strong self-brand connection provokes a positive response from consumers [25,42]. These positive consumer responses (e.g., brand loyalty and willingness to pay a premium) can drive firm performance [13].

Park, MacInnis, and Eisingerich [38] proposed to increase the brand enriching benefits to cultivate customer loyalty behavior. They found that consumers’ perceptions of self-expression lead to the development of loyalty, awareness, and a positive association with brand equity [48]. Customer-based brand equity (CBBE) refers to the ability of consumers to differentiate between brands, as demonstrated in their brand knowledge during marketing communications [15]. Willingness to pay a premium is the primary aspect of brand equity. Consumer WPP can be understood as the additional amount of money that customers are willing to pay for their favorite brand relative to other brands [49]. Thus, the following hypothesis is presented:

**Hypothesis** **(H2).** *BEB positively influences WPP.*

### 3.3. Mediating Role of Brand Happiness

The presence of brand enriching benefits may make consumers happy [43]. Schnebelen and Bruhn [20] proposed that brand happiness is the pleasant experience and spiritual satisfaction that consumers perceive through contact with brands. The cultural meaning and cultural symbolic image of a brand can promote the generation of positive emotions (e.g., happiness) in consumers [35]. The resonance generated by cultural meaning is linked to the consumer psyche [47], and this state of self-harmonizing pleasure and balance is an important antecedent to happiness [50]. Thus, hypothesis 3 is derived as follows:

**Hypothesis** **(H3).** *BEB positively influences brand happiness.*

According to Keller and Lehmann [12], the financial output of a brand comes from the consumer’s mind. In today’s marketplace, consumers not only look for products or services that meet their needs but also want to evoke positive emotions and happiness during use [51]. Boisvert et al. [52] suggest that brand happiness positively influences consumers’ willingness to pay higher prices and that consumers pay higher prices for their favorite brands than for other brands in the same product category in order to gain brand happiness. This psychological phenomenon originates from the equity theory [19,20]. With this, the following hypothesis is presented:

**Hypothesis** **(H4).** *Brand happiness positively influences WPP.*

### 3.4. Moderating Role of Brand Type

Consumer needs vary depending on the brand [53]. Regarding brand types, consumption brands are broadly categorized into functional brands and symbolic brands [54,55]. Symbolic brands are those that reflect a personal identity, allowing consumers to express their personality and identity, enhancing their desired social status [56]. On the other hand, functional brands prioritize practical value and fulfill consumers’ actual needs. For instance, consumers may purchase a house brand soap for handwashing and hygiene improvement. Mitchell and Balabanis [57] argued that differentiating between brand types can lead to a better understanding of consumers’ purchasing intentions in the retail industry.

Previous studies have demonstrated that the brand type moderates consumer behavior [53,55]. The effect of consumer satisfaction on brand loyalty between symbolic and functional brands [55] contrasts the effect of consumer satisfaction on brand loyalty between symbolic and functional brands. Within brand culture, individuals rely on the brand as a medium to establish connections based on their affection for and understanding of the culture [14,44,58]. Both symbolic and functional brands engage with culture during marketing activities. Symbolic brands enable consumers to express their personality and identity, fulfilling their desire for self-brand association [59]. In contrast, functional brands cater to consumers’ utility value interests, lacking inherent self-enrichment for consumers. Consequently, the influence of brand culture on brand equity is more pronounced in functional brands than in symbolic brands.

We posit that brand types will exhibit substantial differences in the relationships among BCI, BEB, and WPP. Specifically, for symbolic brands, the effect of brand culture on WPP will be lower than for functional brands. This leads to hypothesis 5:

**Hypothesis** **(H5).** *Brand types (functional vs. symbolic) moderate the relationship between BCI, BEB, and WPP. Specifically, for functional brands, brand equity resulting from brand culture is higher than for symbolic brands.*

### 3.5. Moderating Role of Perceived Quality

When consumers perceive a brand’s product or service quality to be high, their attitude toward the brand improves [60]. Consumers feel inspired when the brands they purchase and consume incorporate cultural elements they personally love, which can provide enriching benefits [37]. Once consumers establish a personal connection with the brand, a higher quality confers a competitive advantage for the brand and fosters consumer support [49]. Thus, a higher perceived quality amplifies the effect of self-enriching benefits, making consumers more willing to pay a premium [61].

Moreover, in the context of premium retail brands, it is crucial to investigate relevant factors other than quality that can motivate consumers to pay a premium [17]. In this study, we primarily consider the value-added role of brand culture and include perceived quality as a moderating variable in our model. Specifically, a higher perceived brand quality enhances the effect of brand enriching benefits on consumers’ willingness to pay a premium, whereas a lower perceived brand quality weakens this effect. Therefore, based on the existing research, the following hypothesis is presented:

**Hypothesis** **(H6).** *When perceived brand quality is high, it amplifies the effect of brand enriching benefits on consumers’ willingness to pay premium.*

**Figure 1:**
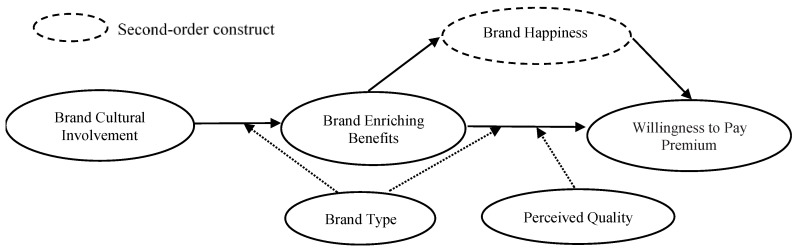
Proposed conceptual model.

Figure 1 presents the research framework of this study.

## 4. Methodology

Our study selected two representative brands from among eight using a pretest. The pretest provided the basis for the preparation of the formal questionnaire and ensured the quality of the questionnaire; then, a formal survey was conducted. In terms of data analysis, the structural equation model (SEM) based on the partial least square (PLS) method was used with SmartPLS (v.3.3.3). This software is suitable for non-normal distributions and multivariable analyses. Additionally, high-order variables are present in this study, so using PLS-SEM is more appropriate.

### 4.1. Pretest

To select the functional and symbolic brands, a pretest (*n* = 70) was conducted based on the method presented by Zhu et al. [62]. First, we provided the participants with definitions and examples of symbolic and functional brands to help them understand the difference between the two. Second, we provided the participants with eight brands (ANTA, Goro’s, Florasis, ZAMANI, Chrome Hearts, To Summer, Chow Tai Fook, and HEA) that utilize cultural elements as well as the definitions of each brand. Finally, we asked them to rank their perception of whether the brand was functional or symbolic (1—functional brands and 2—symbolic brands).

The results of the descriptive analysis showed that the average value of Chow Tai Fook was the highest and that of ANTA was the lowest (M_Chow Tai Fook_ = 1.97, M_ANTA_ = 1.06, M_Goro’s_ = 1.80, M_Florasis_ = 1.33, M_ZAMANI_ = 1.53, M_Chrome Hearts_ = 1.96, M_to summer_ = 1.50, and M_HEA_ = 1.37). We further found a between-subject difference using the significant chi-square test and a one-way ANOVA with SPSS. Chow Tai Fook was different from the remaining four functional brands in terms of consumer-perceived brand type with consumers perceiving Chow Tai Fook as a symbolic brand, despite being a functional brand, alongside HEA, ZAMANI, Florasis, and ANTA. Similarly, ANTA was perceived to be a functional brand, which is different from the other four symbolic brands. The specific results are shown in Table 2. Therefore, we chose ANTA and Chow Tai Fook as the representative functional and symbolic brands, respectively, for this experiment.

### 4.2. Sample and Procedure

A random sample online survey was conducted. Participants were recruited via Credamo (www.credamo.com), which is a professional questionnaire survey website. We randomly assigned 1100 participants, resulting in 570 functional brand participants and 530 symbolic brand participants. The main content of the questionnaire was divided into two parts: the first part included the formal measurement items, while the second involved basic demographic information.

To ensure the quality of the data, specific restrictions were implemented in the questionnaire collection process, such as preventing unique IP addresses and devices from submitting the questionnaire more than once. A total of 1053 usable responses were retained for data analysis after we removed all invalid questionnaires (i.e., questionnaires where all the questions were answered with a single value or were completed too quickly). After checking the reliability of the questionnaire, each participant received CNY 5 (approximately roughly USD 0.80) as a reward for survey completion.

Of the final 1053 participants, the included men (51.66 per cent) and women (48.34 per cent) fell within the following age groups: 18–25 years (15.48 per cent), 26–30 years (19.66 per cent), 31–40 years (37.99 per cent), 41–50 years (18.42 per cent), and 51 and above (8.45 per cent). The educational levels were classified as follows: high school and below (1.52 per cent), college and university degrees (78.45 per cent), and graduate degrees (20.04 per cent).

### 4.3. Measures

Standard and validated measurement scales were selected for use based on a literature review. Brand cultural involvement (BCI) was measured with a five-item scale adapted from Whang, Yong, and Ko [11]. Next, a three-item brand enriching benefit (BEB) scale from Park, B. Eisingerich, and Park [43] and Oh, Prado, Korelo, and Frizzo [39] was used. The brand happiness scale was measured using a 12-item scale adapted from Mansoor and Paul [22], and Schnebelen and Bruhn [20]. Willingness to pay a premium (WPP) was measured using a three-item scale adapted from Sarkar et al. [63]. Perceived quality (PQ) was measured using the four-item scale from Parris and Guzmán [49]. All scale items are reported in Table 3 and were measured on a seven-point Likert scale (1 = strongly disagree, 7 = strongly agree).

## 5. Data Analysis and Results

### 5.1. Measurement Model Analysis

The measurement model had good fit (SRMR = 0.05 < 0.08, NFI = 0.848) [64]. The model exhibited no collinearity problems, and the proposed framework had a strong ability to predict BEB (R^2^ = 0.505), BH (R^2^ = 0.576), and WPP (R^2^ = 0.621) (R^2^; >0.67 is acceptable for practical use and >0.5 is moderately acceptable). The model also had good predictive ability, as indicated by a high predictive relevance (Q^2^) that was obtained through blindfolding [65], BEB (Q^2^ = 0.282), BH (Q^2^ = 0.320), and WPP (Q^2^ = 0.450).

The items also had good internal consistency with Cronbach’s alpha (α) values > 0.7. The validity of the model contains convergent and discriminant validity. Convergent validity refers to the degree to which the item is related to the variable, which could be assessed by the average variance extracted (AVE) and the composite reliability (CR). In our study, the AVE of all latent variables was greater than the requisite minimum of 0.5 (see Table 3), indicating that the scale had good convergent validity [66].

The scale also had good discriminant validity. Discriminant validity refers to the degree of differentiation between different variables. This is evaluated by comparing the square root of AVE and the size of the correlation coefficient (see Table 4). Additionally, the HTMT values of all indicators in this study are lower than 0.9, which presents a good discriminant validity. In addition, various procedural solutions were implemented to control common method variance (CMV). First, we ensured anonymity and let respondents know that there were no right or wrong answers [24]. The CMV was initially assessed using the Harman’s single-factor test, where the critical value of the first factor was statistically less than 50% [67]. The results revealed that only 44% of the total variance (<50%) derived from a single factor, indicating no common method bias. Finally, all VIFs in our inner model received values lower than 3.3 on a full collinearity test, suggesting that common method bias did not affect the data and indicating no multicollinearity problems [64,66].

### 5.2. Structural Model Analysis

The results for the hypotheses are as follows (see Table 5). Firstly, BCI positively influences BEB (β = 0.604; *t* = 22.481; *p* < 0.001). Therefore, H1 was supported. This indicates that the brands have cultural elements that consumers personally love, which can bring about brand enriching benefits. Secondly, BEB positively influences WPP (β = 0.283; *t* = 6.446; *p* < 0.001). H2 was thus supported. Brand enriching benefits can positively influence consumers’ willingness to pay a premium. WPP, as a key indicator of brand equity, reflects a brand’s ability to command a higher price than its competitors, while brand culture can enhance competitiveness for brands. Then, the mediating effect of brand happiness was examined. BEB positively influences BH (β = 0.758; *t* = 32.334; *p* < 0.001), and BH positively influences WPP (β = 0.442; *t* = 9.07; *p* < 0.001). Thus, H3 was supported. Boisvert, Christodoulides, and Sajid Khan [52] argued that consumers are willing to make monetary sacrifices to obtain happiness. The results of this mediating effect support this idea. Brand enriching benefits make consumers happy, and they are willing to make monetary sacrifices to obtain brand happiness. Brand managers could give more consideration to consumer happiness when shaping the association between brand and culture in the brand experience.

### 5.3. Moderating Effect of Brand Type and Perceived Quality

In order to test the moderating effect of brand type (H5), a multi-group analysis was used. Both groups showed good internal consistency and scale reliability [68] (see Table 3). Cheah et al. [69] demonstrated the need to test the measurement invariance of composites using partial least squares. Invariance tests were conducted on the two data sets separately before comparing the groups. In this study, the invariance test was conducted by running the MICOM procedure in PLS, and the results are shown in Table 6. First, in both groups, the latent variable measurements were scored on a seven-point scale, and the topics remained consistent; that is, configural invariance was satisfied. In step 2, the model was shown to satisfy compositional invariance, which means that the model of the relationship between the constructs in each group was the same. With this, the conditions for conducting a multi-group analysis were satisfied, and the path coefficients between the groups were compared. In step 3, basically no difference was found in the means between the constructs. Therefore, the model satisfies partial invariance [70].

Firstly, the respondents were split into two groups: 550 functional brand participants and 503 symbolic participants. Through a multi-group analysis, in the functional brand group, BCI had a greater effect on BEB than in the symbolic brand group (correlation coefficient: β_functional_ = 0.778, β_symbolic_ = 0.449). Consistent with this trend, in the functional brand group, BEB had a greater effect on WPP than in the symbolic brand group (correlation coefficient: β_functional_ = 0.438, β_symbolic_ = 0.19) (see Table 7). Thus, H5 is supported. Previous research has shown that brand type moderates consumer behavior [53,55]. The findings of this study suggest a distinction between emotional and functional brands also in the brand culture’s influence on brand equity. Specifically, purchasing a functional brand’s product, such as ANTA sneakers, creates more associations as well as a willingness to pay a premium if it has an associated cultural meaning, because such a purchase fulfills multiple customer needs. A key reason why the proposed effects are stronger for functional brands is that functional brands leave more room for brand culture to enrich their meanings. Functional brands, like ANTA, fulfill multiple consumer needs, such as practicality and performance. When cultural meaning is attached to these products, it enhances their value by creating deeper associations related to identity, community, etc. For example, owning ANTA sneakers may not only meet performance needs but also represent pride in the local craftsmanship or national identity, increasing consumers’ willingness to pay a premium.

For symbolic brands, consumers can express their personality and identity, which already satisfies consumers’ interest in the self-brand association [59]. Therefore, the influence of brand culture on brand equity will be weaker in symbolic brands than in functional brands. Brand managers can focus on the difference between brand types themselves when applying culture to brand identity, product design, etc.

We introduced the interactive items brand enriching benefit and perceived quality into the model, and the results indicate that perceived quality had a significant moderating effect on the relationship between brand enriching benefit and consumers’ willingness to pay a premium (β = 0.068, *t* = 3.067, *p* < 0.05).

**Figure 2:**
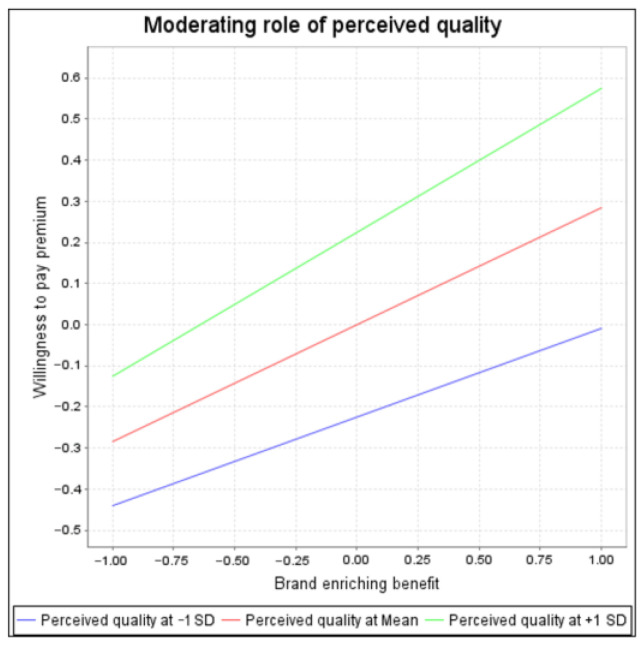
Moderating effect of high, medium, and low values of perceived quality.

The simple slope test depicted in Figure 2 illustrates the moderating effect at high, medium, and low values. The slope for a high level of perceived quality was higher than that of for a low level, indicating that when consumers perceive a high degree of quality, the relationship between brand enriching benefit and consumers’ willingness to pay a premium will be strengthened. In the context of premium retail brands, it is crucial to investigate relevant factors other than quality that can motivate consumers to pay a premium [17]. We considered perceived quality as a moderating variable in this model. Davcik and Sharma [61] indicated that better quality would result in a person willing to pay the higher prices. Our research indicates that the rich benefits of a brand may bring about brand equity. At the same time, perceived quality plays a positive moderating role in the relationship between brand enriching benefits and brand equity.

## 6. Discussion

### 6.1. Summary of Findings

Empirical results demonstrate that consumers’ involvement in cultural meaning is beneficial in generating strong brand enriching benefits, which evokes a positive response from consumers and improves their willingness to pay a premium. This process reflects the value added from the brand culture to the brand. The brand enriching benefits are the key factor [37]. The self-brand association that results from consumers’ affection for the culture is an important antecedent of brand equity.

In our study of the mediating effects, we found that brand culture triggers consumers’ willingness to pay a premium through brand enriching benefits and happiness, and brand happiness is an important predictor of consumers’ WPP for brand culture. These results are consistent with those of Park, Eisingerich, and Park [43] that the presence of brand enriching benefits may make consumers happy. In order to obtain brand happiness, consumers pay a higher price for their favorite brand instead of other brands in the same product category [19,20], promoting brand equity.

Through a multi-group analysis, we found that for functional brands, brand culture has a greater effect on consumers’ willingness to pay a premium than for symbolic brands. This is because consumers purchase functional brands to satisfy their utility needs, while symbolic brands can be used to express their personality and identity and already satisfy their self-branding needs [59]. The results of the current study also indicate the moderating role of perceived quality. A higher perceived quality of a brand can effectively contribute to the brand enriching benefits and consumers’ willingness to pay a premium. Therefore, brand managers should pay attention to product quality and brand type when adding cultural elements in their brand experience.

### 6.2. Theoretical Implications

This research contributes to the literature on brand culture and enriches brand culture theory. The findings of this study show that brand culture can add value to a brand, which is in line with what most scholars have mentioned [29,32,46], but this study fills the research gap on how brand culture can bring value to a brand. A previous study also advocated for adding brand enriching benefits to cultivate customer loyalty behavior [37], and their empirical results demonstrated that consumers’ involvement with culture can lead to brand enriching benefits, which impacts consumers’ willingness to pay a premium.

Moreover, this study extends the study of equity theory in the context of brand culture. Consumers pay higher prices for their favorite brands over other brands in the same product category in order to obtain brand happiness [52], which is a psychological phenomenon derived from equity theory [19,20]. Previous studies mentioned that brand love is a significant predictor of consumers’ WPP for purchasing a loved brand as well as consumers’ willingness to make monetary and/or non-monetary sacrifices for a loved brand [71]. The presence of brand enriching benefits may make consumers happy [43]. In this study, brand happiness was used as a mediating variable, where consumers’ brand self-association due to cultural recognition and affection for the brand influenced brand equity through brand happiness.

Previous studies have shown that brand type has a moderating effect on consumer behavior [53,55]. We extended current research on brand type in brand culture by investigating the moderating role of brand type. Instead of focusing on a single brand type, this study investigated the differences in the formation of brand culture in two classic brand types. The results show that the effect of brand culture on brand equity is stronger in functional brands and weaker in symbolic brands with a significant difference between the two types, supporting H5. Therefore, brand managers should consider differentiating brand type levels when launching products.

### 6.3. Practical Implications

The results propose specific strategies and new ideas for managers in building brand culture and accumulating brand equity. Furthermore, brands can strengthen the cultural meaning of their brands. It is crucial for managers to use culture and its rich benefits wisely. For example, they can find appropriate cultural values to serve as the core of their marketing communications, refine cultural elements, and apply them innovatively. Brands can extract elements from traditional culture that are relevant to and representative of them, such as characters, patterns, and colors, and apply them to brand identity, product design, marketing strategy, etc. In this way, they shape the association between brand and culture in the brand experience [29]. Managers are able to transfer cultural meanings into the brand, and consumers are able to facilitate the process of cultural meaning transfer [32].

Another significant finding of this study is that consumers’ affection for a culture contributes more to brand equity in functional brands than in symbolic brands. ANTA, a leading Chinese sportswear brand, exemplifies the functional brand by prioritizing practicality, performance, and utilitarian value in its products. Functional brands, like ANTA, emphasize product performance, durability, and usability—attributes that are central to many brands in industries such as the electronics and home appliance industries. This makes ANTA a fitting representative of functional brands, where product utility is at the forefront of its brand identity. Chow Tai Fook, on the other hand, is a well-known luxury jewelry brand that symbolizes emotional and social value, aligning with the attributes of a symbolic brand. Symbolic brands, like Chow Tai Fook, focus on identity expression and emotional appeal, which are characteristic of brands in luxury fashion, premium accessories, and high-end consumer goods. Our findings are relevant for marketers and brand strategists working with a variety of brands that fall into these categories. Brand managers can leverage these insights by recognizing the differences between brand types when applying cultural elements to the design of brand avatars, brand logos, social media marketing, and products [25,72,73] so that consumers can feel the association between the brand and their culture in the brand experience. For symbolic brands, efforts may be better focused on reinforcing existing emotional or aspirational associations rather than heavily relying on cultural integration. Functional brands leave more room for brand culture to enrich their meanings. Managers can significantly benefit from incorporating cultural elements into their brand identity, as it can deepen consumer connections and increase brand equity. Perceived quality, as a moderating factor, also plays a contributing role in the BEB-WPP process. In alignment with this finding, a commitment to ensuring a higher-perceived brand quality is beneficial to brand equity.

On the other hand, managers must consider consumer happiness in the brand experience. Consumers are likely to build a long-term relationship with brands if they feel a sense of happiness from an absorptive environment that induces flow experience [50]. The building of a brand culture benefits consumers and society in addition to providing economic benefits to the business itself, as it helps to generate positive emotions. In addition, brand happiness influences the willingness of consumers to make more monetary sacrifices for a favorite brand culture, which is an ideal prospect for brand managers who want to build strong brands. Managers can establish brand communities to inquire about and record consumer happiness levels.

### 6.4. Limitations and Future Research

There are several limitations in this study, which can be further studied and discussed in the future. A limitation of this study is the lack of distinction between different cultural cues. Some scholars have distinguished local culture from global culture [74]. For example, compared with iconic authenticity cues (i.e., real replicas of the same brand), index authenticity cues (i.e., original products of the brand) lead to greater brand moral perception [75]. The effects of iconic and index authenticity characteristics on consumption behaviors are issues worthy of further exploration.

From the results of self-reports and anonymous questionnaires, our survey concludes that culture affects consumer willingness to pay a premium for brands. However, the specific premium range and pricing strategy of products have not been sufficiently developed, which is another limitation of this study. Future research can further expand upon these findings by combining economic methods and formulating product pricing strategies for enterprises.

## Figures and Tables

**Table 1:** The existing literature on brand culture.

Author(s) and Year	Main Focus		Methodology
	Determinants	Consequences	
Holt (2002) [4]	Brand culture comes from historical deposits, a collection of relevant stories told by corporate history, mainstream culture, and customers.		Qualitative research
Banerjee (2008) [5]	A Brand–Culture Fit Model was constructed, proposing to combine brand heritage and a country’s cultural heritage to find commonalities in order to build brand culture.		Qualitative research
Park and Rabolt (2009) [26]		Cultural value is identified as an influential factor on brand image and consumer consumption value.	Qualitative research
Wiedmann et al. (2011) [14]		The cultural value of a brand is taken as an important component in the concept of brand heritage.	Empirical research
Torelli and Ahluwalia (2012) [27]		Cultural congruity between a brand and a product can influence extension evaluations over and above perceptions of fit.	Empirical research
Schembri and Latimer (2016) [7]	Brand culture in online brand communities is co-created by community members in a variety of ways.		Qualitative research
He and Wang (2017) [28]		The cultural compatibility of global brands that incorporate Chinese cultural elements has a direct positive impact on consumers’ purchasing likelihood.	Empirical research
Fournier and Alvarez (2019) [29]	How are cultural meanings integrated into the brand, and how are cultural models used to understand the brand experience in their daily lives?		Qualitative research
Porcu et al. (2020) [30]		The relationship between culture and brand performance is confirmed from a corporate culture perspective.	Empirical research
Our study	How do brands gain cultural meaning and promote consumers’ willingness to pay a premium? What are the underlying mechanisms (moderating and mediating effects) of the relationship?		

**Table 2:** Between-subject differences by brand type.

Brands	Brands	Differences in the Average Values	SD	*p* Value	95% CI	
					LLCI	ULCI
Chow Tai Fook	HEA	0.600	0.062	<0.001	0.40	0.80
	ZAMANI	0.443	0.063	<0.001	0.24	0.65
	Florasis	0.643	0.060	0.000	0.45	0.84
	ANTA	0.914	0.034	0.000	0.80	1.02
ANTA	Goro’s	−0.743	0.056	0.000	−0.92	−0.57
	Chow Tai Fook	−0.914	0.034	0.000	−1.02	−0.80
	Chrome Hearts	−0.900	0.037	0.000	−1.02	−0.78
	to summer	−0.443	0.066	<0.001	−0.66	−0.23

**Table 3:** Measurement scales and results on outer loading, construct reliability, and validity.

Construct and Their Items	Full Sample	Functional Brands	Symbolic Brands
Brand Enriching Benefit (BEB)	α = 0.857; CR = 0.913; AVE = 0.778	α = 0.865; CR = 0.918; AVE = 0.788	α = 0.848; CR = 0.908; AVE = 0.767
To what extent does this brand misrepresent or represent who you are as a person.	0.874	0.878	0.871
To what extent does this brand suppress or express who you want to be.	0.875	0.879	0.871
To what extent does this brand undermine or reinforce your values.	0.897	0.905	0.886
Brand Happiness (BH)	α = 0.929, CR = 0.939, AVE = 0.563	α = 0.944, CR = 0.951, AVE = 0.619	α = 0.902, CR = 0.918, AVE = 0.598
Joy (α = 0.872, CR = 0.922, AVE = 0.797)		α = 0.898	α = 0.820
BH1: I feel glad buying/using this brand.	0.890	0.909	0.857
BH2: I feel cheerful buying/using this brand.	0.877	0.898	0.835
BH3: I feel joyful buying/using this brand.	0.911	0.927	0.879
Vigor (α = 0.858, CR = 0.913, AVE = 0.779)		α = 0.887	α =0.817
BH4: Buying/using this brand makes me feel lively.	0.871	0.894	0.837
BH5: Buying/using this brand makes me feel peppy.	0.885	0.907	0.857
BH6: Buying/using this brand makes me feel vigorous.	0.891	0.909	0.872
Pride (α = 0.854, CR = 0.911, AVE = 0.774)		α = 0.884	α = 0.805
BH7: Buying/using this brand makes me feel proud.	0.871	0.892	0.840
BH8: Buying/using this brand makes me feel superior.	0.875	0.897	0.842
BH9: Buying/using this brand makes me feel worthy.	0.893	0.914	0.862
Serenity (α = 0.870, CR = 0.920, AVE = 0.793)		α = 0.892	α = 0.835
BH10: Buying/using this brand makes me feel relaxed.	0.893	0.908	0.868
BH11: I feel myself at ease buying/using this brand.	0.895	0.909	0.877
BH12: Buying/using this brand makes me feel comfortable.	0.885	0.904	0.856
Brand Cultural Involvement (BCI)	α = 0.907, CR = 0.931, AVE = 0.728	α = 0.907, CR = 0.931, AVE = 0.728	α = 0.909, CR = 0.933, AVE = 0.735
Love and enjoy Chinese culture.	0.843	0.828	0.861
Fan of Chinese culture.	0.825	0.858	0.806
More concerned with Chinese culture.	0.859	0.863	0.851
Interested in Chinese culture.	0.873	0.859	0.886
Closeness to Chinese culture.	0.867	0.859	0.879
Perceived Quality (PQ)	α = 0.860, CR = 0.905, AVE = 0.704	α = 0.856, CR = 0.902, AVE = 0.698	α = 0.866, CR = 0.908, AVE = 0.713
Compared with other brands of (product), (brand name) is of very high quality.	0.800	0.782	0.828
(Brand name) is the best brand in its product class.	0.859	0.870	0.844
(Brand name) consistently performs better than all other brands of (product).	0.867	0.865	0.867
I can always count on (brand name) brand of (product) for consistent high quality.	0.829	0.821	0.837
Willingness to Pay Premium (WPP)	α = 0.820, CR = 0.893, AVE = 0.738	α = 0.841, CR = 0.905, AVE = 0.761	α = 0.787, CR = 0.877; AVE = 0.705
I am willing to pay a higher price for brand X of (product) than for other brands of (product).	0.910	0.914	0.902
The price of brand X would have to increase quite a bit before I would switch to another brand of (product).	0.751	0.778	0.722
I am willing to pay a lot more for brand X than other brands of (product category)	0.906	0.918	0.884

All items were measured on 7-point Likert scale. α = Cronbach’s alpha.

**Table 4:** Discriminant validity (Fornell–Larcker criterion).

	BCI	BEB	Joy	PQ	Pride	Serenity	Vigor	WPP
BCI	**0.853**							
BEB	0.604	**0.882**						
Joy	0.549	0.648	**0.893**					
PQ	0.552	0.731	0.603	**0.839**				
Pride	0.522	0.668	0.616	0.588	**0.880**			
Serenity	0.501	0.593	0.645	0.555	0.581	**0.891**		
Vigor	0.543	0.656	0.620	0.581	0.636	0.633	**0.882**	
WPP	0.580	0.698	0.667	0.651	0.633	0.588	0.632	**0.869**

BCI: brand cultural involvement; BEB: brand enriching benefit; PQ: perceived quality; WPP: willingness to pay premium. The bold characters on the diagonal are the square root of AVE, and the values below the diagonal line are the correlations.

**Table 5:** Hypothesis test results.

Hypothesis Path	Original Sample (O)	Standard Deviation (STDEV)	*t* Statistics	*p* Values	Results
H1: BCI → BEB	0.604	0.027	22.481	0.000	Accepted
H2: BEB → WPP	0.283	0.044	6.446	0.000	Accepted
H3: BEB → BH	0.758	0.023	32.334	0.000	Accepted
H4: BH → WPP	0.442	0.049	9.07	0000	Accepted
BCI → BEB → WPP	0.171	0.029	5.852	0.000	Accepted
BCI → BEB → BH	0.458	0.031	14.643	0.000	Accepted
BCI → BEB → BH → WPP	0.203	0.024	8.269	0.000	Accepted
BEB → BH → WPP	0.335	0.038	8.882	0.000	Accepted

**Table 6:** Results of invariance tests using permutation.

Construct	Configural Invariance (Step1)	Compositional Invariance (Step2)		Partial Measurement Invariance	Equal Mean Assessment (Step3)		
		Original Correlation	5%		Mean—Original Difference	Permutation *p*-Values	Confidence Interval
BCI	Yes	1.000	1	Yes	0.057	0.32	[−0.12, 0.11]
BEB	Yes	1.000	1	Yes	−0.008	0.87	[−0.12, 0.107]
BH	Yes	1.000	1	Yes	−0.081	0.176	[−0.123, 0.109]
Joy	Yes	1.000	1	Yes	−0.179	0.008	[−0.114, 0.107]
PQ	Yes	1.000	0.999	Yes	0.016	0.792	[−0.112, 0.102]
Pride	Yes	1.000	1	Yes	−0.08	0.182	[−0.119, 0.111]
Serenity	Yes	1.000	1	Yes	0	1	[−0.131, 0.12]
Vigor	Yes	1.000	1	Yes	−0.01	0.858	[−0.125, 0.116]
WPP	Yes	1.000	0.999	Yes	−0.083	0.148	[−0.109, 0.107]

**Table 7:** Moderated effect analysis of brand type (partial least squares-based multi-group analysis).

Hypothesis Path	Functional (*n* = 550)			Symbolic (*n* = 503)			Difference between Coefficient	
	Path Coefficient	Standard Deviation	*t*-Value	Path Coefficient	Standard Deviation	*t*-Value	Path Coefficient	*p* Value
BCI → BEB	0.778	0.025	31.434	0.449	0.042	10.61	0.33	0.000
BEB → WPP	0.438	0.058	7.527	0.19	0.06	3.161	0.248	0.003

## Data Availability

The data supporting the findings of this study are available from the corresponding author upon reasonable request.

## References

[1] Southworth S.S. (2019). U.S. Consumers’ Perception of Asian Brands’ Cultural Authenticity and Its Impact on Perceived Quality, Trust, and Patronage Intention. J. Int. Consum. Mark..

[2] Torelli C.J., Stoner J.L. (2016). Managing cultural equity: A theoretical framework for building iconic brands in globalized markets. Rev. Mark. Res..

[3] Carlgren L., BenMahmoud-Jouini S. (2022). When cultures collide: What can we learn from frictions in the implementation of design thinking?. J. Prod. Innov. Manag..

[4] Holt D.B. (2002). Why Do Brands Cause Trouble? A Dialectical Theory of Consumer Culture and Branding. J. Consum. Res..

[5] Banerjee S. (2008). Strategic Brand-Culture Fit: A conceptual framework for brand management. J. Brand Manag..

[6] Healy J.C., McDonagh P. (2013). Consumer roles in brand culture and value co-creation in virtual communities. J. Bus. Res..

[7] Schembri S., Latimer L. (2016). Online brand communities: Constructing and co-constructing brand culture. J. Mark. Manag..

[8] Moorlock E., Dekel-Dachs O., Stokes P., Larsen G. (2023). Constructing Consumer-Masstige brand relationships in a volatile social reality. J. Bus. Res..

[9] Havitz M.E., Mannell R.C. (2005). Enduring involvement, situational involvement, and flow in leisure and non-leisure activities. J. Leis. Res..

[10] Guo Y., Cao Z., Zhu Z. (2022). The influence of ICH-narrator/self-congruity on tourist’s purchase intention of intangible cultural heritage products in a narrative context. J. Hosp. Tour. Manag..

[11] Whang H., Yong S., Ko E. (2016). Pop culture, destination images, and visit intentions: Theory and research on travel motivations of Chinese and Russian tourists. J. Bus. Res..

[12] Keller K.L., Lehmann D.R. (2003). How do brands create value?. Mark. Manag..

[13] Bairrada C.M., Coelho F., Coelho A. (2018). Antecedents and outcomes of brand love: Utilitarian and symbolic brand qualities. Eur. J. Mark..

[14] Wiedmann K.-P., Hennigs N., Schmidt S., Wuestefeld T. (2011). Drivers and outcomes of brand heritage: Consumers’ perception of heritage brands in the automotive industry. J. Mark. Theory Pract..

[15] Keller K.L. (1993). Conceptualizing, Measuring, and Managing Customer-Based Brand Equity. J. Mark..

[16] Casidy R., Wymer W. (2016). A risk worth taking: Perceived risk as moderator of satisfaction, loyalty, and willingness-to-pay premium price. J. Retail. Consum. Serv..

[17] Fatma M., Khan I. (2024). Brand authenticity and consumers’ willingness to pay a premium price (WPP): The mediating role of brand identification. J. Brand Manag..

[18] Kumar V., Kaushik A.K. (2022). Engaging customers through brand authenticity perceptions: The moderating role of self-congruence. J. Bus. Res..

[19] Homburg C., Koschate N., Hoyer W.D. (2005). Do satisfied customers really pay more? A study of the relationship between customer satisfaction and willingness to pay. J. Mark..

[20] Schnebelen S., Bruhn M. (2018). An appraisal framework of the determinants and consequences of brand happiness. Psychol. Mark..

[21] Albert N., Merunka D., Valette-Florence P. (2013). Brand passion: Antecedents and consequences. J. Bus. Res..

[22] Mansoor M., Paul J. (2022). Mass prestige, brand happiness and brand evangelism among consumers. J. Bus. Res..

[23] Bae B.R., Kim S.-E. (2023). Effect of brand experiences on brand loyalty mediated by brand love: The moderated mediation role of brand trust. Asia Pac. J. Mark. Logist..

[24] Dwivedi A., Nayeem T., Murshed F. (2018). Brand experience and consumers’ willingness-to-pay (WTP) a price premium: Mediating role of brand credibility and perceived uniqueness. J. Retail. Consum. Serv..

[25] Samarah T., Bayram P., Aljuhmani H.Y., Elrehail H. (2022). The role of brand interactivity and involvement in driving social media consumer brand engagement and brand loyalty: The mediating effect of brand trust. J. Res. Interact. Mark..

[26] Park H.-J., Rabolt N.J. (2009). Cultural value, consumption value, and global brand image: A cross-national study. Psychol. Mark..

[27] Torelli C.J., Ahluwalia R. (2012). Extending Culturally Symbolic Brands: A Blessing or a Curse?. J. Consum. Res..

[28] He J., Wang C.L. (2017). How global brands incorporating local cultural elements increase consumer purchase likelihood. Int. Mark. Rev..

[29] Fournier S., Alvarez C. (2019). How brands acquire cultural meaning. J. Consum. Psychol..

[30] Porcu L., del Barrio-García S., Kitchen P.J., Tourky M. (2020). The antecedent role of a collaborative vs. a controlling corporate culture on firm-wide integrated marketing communication and brand performance. J. Bus. Res..

[31] Hungara A., Nobre H. (2021). A consumer culture theory perspective of the marketplace: An integrative review and agenda for research. Int. J. Consum. Stud..

[32] McCracken G. (1986). Culture and Consumption: A Theoretical Account of the Structure and Movement of the Cultural Meaning of Consumer Goods. J. Consum. Res..

[33] Steenkamp J.-B.E. (2019). Global versus local consumer culture: Theory, measurement, and future research directions. J. Int. Mark..

[34] Gürhan-Canli Z., Sarial-Abi G., Hayran C. (2018). Consumers and brands across the globe: Research synthesis and new directions. J. Int. Mark..

[35] Jian Y., Zhou Z., Zhou N. (2019). Brand cultural symbolism, brand authenticity, and consumer well-being: The moderating role of cultural involvement. J. Prod. Brand Manag..

[36] Molinillo S., Japutra A., Ekinci Y. (2022). Building brand credibility: The role of involvement, identification, reputation and attachment. J. Retail. Consum. Serv..

[37] Jun M., Han J., Zhou Z., Eisingerich A.B. (2023). When is celebrity endorsement effective? Exploring the role of celebrity endorsers in enhancing key brand associations. J. Bus. Res..

[38] Park C.W., MacInnis D.J., Eisingerich A.B. (2016). Brand Admiration: Building a Business People Love.

[39] Oh H., Prado P.H.M., Korelo J.C., Frizzo F. (2019). The effect of brand authenticity on consumer–brand relationships. J. Prod. Brand Manag..

[40] Richins M.L. (1994). Special Possessions and the Expression of Material Values. J. Consum. Res..

[41] Yeh C.-H., Wang Y.-S., Yieh K. (2016). Predicting smartphone brand loyalty: Consumer value and consumer-brand identification perspectives. Int. J. Inf. Manag..

[42] Bilro R.G., Loureiro S.M.C. (2023). I am feeling so good! Motivations for interacting in online brand communities. J. Res. Interact. Mark..

[43] Park C.W., Eisingerich A.B., Park J.W. (2013). Attachment–aversion (AA) model of customer–brand relationships. J. Consum. Psychol..

[44] Rose G.M., Merchant A., Orth U.R., Horstmann F. (2016). Emphasizing brand heritage: Does it work? And how?. J. Bus. Res..

[45] Liu H., Schoefer K., Fastoso F., Tzemou E. (2021). Perceived Brand Globalness/Localness: A Systematic Review of the Literature and Directions for Further Research. J. Int. Mark..

[46] Batra R. (2019). Creating Brand Meaning: A Review and Research Agenda. J. Consum. Psychol..

[47] Price L.L., Coulter R.A. (2019). Crossing bridges: Assembling culture into brands and brands into consumers’ global local cultural lives. J. Consum. Psychol..

[48] Salem S.F., Alanadoly A.B., Sulaiman M.A.B.A. (2024). Immersive gaming in the fashion arena: An investigation of brand coolness and its mediating role on brand equity. J. Res. Interact. Mark..

[49] Parris D.L., Guzmán F. (2023). Evolving brand boundaries and expectations: Looking back on brand equity, brand loyalty, and brand image research to move forward. J. Prod. Brand Manag..

[50] Huh J., Kim H.-Y., Lee G. (2023). “Oh, happy day!” Examining the role of AI-powered voice assistants as a positive technology in the formation of brand loyalty. J. Res. Interact. Mark..

[51] Kumar A., Paul J., Starčević S. (2021). Do brands make consumers happy? A masstige theory perspective. J. Retail. Consum. Serv..

[52] Boisvert J., Christodoulides G., Sajid Khan M. (2023). Toward a better understanding of key determinants and consequences of masstige consumption. J. Bus. Res..

[53] Kim S.J., Yoo J., Ko E. (2023). The effects of brand collaboration with the online game on customer equity and purchase intention: Moderating effect of fashion brand type. Asia Pac. J. Mark. Logist..

[54] Bhat S., Reddy S.K. (1998). Symbolic and Functional Positioning of Brands. J. Consum. Mark..

[55] Fernandes T., Moreira M. (2019). Consumer brand engagement, satisfaction and brand loyalty: A comparative study between functional and emotional brand relationships. J. Prod. Brand Manag..

[56] Fuentes H., Vera-Martinez J., Kolbe D. (2023). The role of intangible attributes of luxury brands for signalling status: A systematic literature review. Int. J. Consum. Stud..

[57] Mitchell V.W., Balabanis G. (2021). The role of brand strength, type, image and product-category fit in retail brand collaborations. J. Retail. Consum. Serv..

[58] Peng L., Xie T. (2016). Making similarity versus difference comparison affects perceptions after bicultural exposure and consumer reactions to culturally mixed products. J. Cross-Cult. Psychol..

[59] Escalas J.E., Bettman J.R. (2005). Self-construal, reference groups, and brand meaning. J. Consum. Res..

[60] Lin Y.-H., Lin F.-J., Wang K.-H. (2021). The effect of social mission on service quality and brand image. J. Bus. Res..

[61] Davcik N.S., Sharma P. (2015). Impact of product differentiation, marketing investments and brand equity on pricing strategies: A brand level investigation. Eur. J. Mark..

[62] Zhu X., Teng L., Foti L., Yuan Y. (2019). Using self-congruence theory to explain the interaction effects of brand type and celebrity type on consumer attitude formation. J. Bus. Res..

[63] Sarkar J.G., Sreejesh S., Sarkar A., Dwivedi Y.K. (2021). Impact of self-brand connection on willingness to pay premium: Relevant mediators and moderators. Psychol. Mark..

[64] Blanco-Moreno S., Costa-Feito A., Santos C.R., González-Fernández A.M. (2024). Women’s happiness and brand content marketing. Manag. Decis..

[65] Masuda H., Han S.H., Lee J. (2022). Impacts of influencer attributes on purchase intentions in social media influencer marketing: Mediating roles of characterizations. Technol. Forecast. Soc. Change.

[66] Becker J.-M., Cheah J.-H., Gholamzade R., Ringle C.M., Sarstedt M. (2023). PLS-SEM’s most wanted guidance. Int. J. Contemp. Hosp. Manag..

[67] Zafar A.U., Shen J., Ashfaq M., Shahzad M. (2021). Social media and sustainable purchasing attitude: Role of trust in social media and environmental effectiveness. J. Retail. Consum. Serv..

[68] Leitgöb H., Seddig D., Asparouhov T., Behr D., Davidov E., De Roover K., Jak S., Meitinger K., Menold N., Muthén B. (2023). Measurement invariance in the social sciences: Historical development, methodological challenges, state of the art, and future perspectives. Soc. Sci. Res..

[69] Cheah J.-H., Amaro S., Roldán J.L. (2023). Multigroup analysis of more than two groups in PLS-SEM: A review, illustration, and recommendations. J. Bus. Res..

[70] Kadic-Maglajlic S., Arslanagic-Kalajdzic M., Micevski M., Dlacic J., Zabkar V. (2019). Being engaged is a good thing: Understanding sustainable consumption behavior among young adults. J. Bus. Res..

[71] Palusuk N., Koles B., Hasan R. (2019). ‘All you need is brand love’: A critical review and comprehensive conceptual framework for brand love. J. Mark. Manag..

[72] Foster J.K., McLelland M.A., Wallace L.K. (2022). Brand avatars: Impact of social interaction on consumer–brand relationships. J. Res. Interact. Mark..

[73] Elsharnouby M.H., Jayawardhena C., Liu H., Elbedweihy A.M. (2023). Strengthening consumer–brand relationships through avatars. J. Res. Interact. Mark..

[74] Nie C., Wang T. (2021). How global brands incorporate local cultural elements to improve brand evaluations: A perspective on cultural mixing. Int. Mark. Rev..

[75] Kerviler G.d., Heuvinck N., Gentina E. (2021). “Make an Effort and Show Me the Love!” Effects of Indexical and Iconic Authenticity on Perceived Brand Ethicality. J. Bus. Ethics.

